# Opiate maintenance patients’ attitudes and self-reported adherence to protective measures against SARS-CoV-2 infections

**DOI:** 10.3389/fpsyt.2023.1254053

**Published:** 2023-09-14

**Authors:** Michael Specka, Tobias Kröckert, Mirko Trilling, Udo Bonnet, Fabrizio Schifano, Norbert Scherbaum

**Affiliations:** ^1^Department of Psychiatry and Psychotherapy, LVR-Hospital Essen, Medical Faculty, University of Duisburg-Essen, Essen, Germany; ^2^Institute for Virology, University Hospital Essen, University of Duisburg-Essen, Essen, Germany; ^3^Department of Psychiatry, Psychotherapy and Psychosomatic Medicine, Evangelisches Krankenhaus Castrop-Rauxel, Academic Teaching Hospital of the University of Duisburg-Essen, Essen, Germany; ^4^Psychopharmacology, Drug Misuse and Novel Psychoactive Substances Research Unit, School of Life and Medical Sciences, University of Hertfordshire, Hertfordshire, United Kingdom

**Keywords:** opioid maintenance treatment, COVID-19 pandemic, somatic comorbidity, protective measures, vaccination rate

## Abstract

**Background:**

During the COVID-19 pandemic we assessed to which extent patients in opioid maintenance treatment (OMT) adhere to official recommendations regarding preventive intervention strategies against COVID-19.

**Methods:**

Patients enrolled in two OMT clinics in Germany were interviewed applying a standardized questionnaire, which covered socio-demographic information, recent psychotropic substance use, recent social activities, the history of SARS-CoV-2 infection, attitudes toward official protection recommendations, and levels of adherence to these suggestions. Current mental and medical diagnoses were retrieved from medical files. In subjects without known infection and without vaccination, blood samples were tested for the identification of anti-SARS-CoV-2-S-antibodies. Interviews were performed between the end of May and the end of September 2021.

**Results:**

Patients’ (*n* = 155) average age was 47 years; 74% were males. In addition to the opiate dependence, in nearly 80% of cases another medical disorder was recorded. The range of medical factors that predispose for severe COVID-19 outcomes were present in 39% of patients; 18% of the sample refused to be vaccinated. Nearly all patients reported having carried out a range of activities outside their residence during the week prior to the interviews, including visits of treatment facilities (86.5%; 95% confidence interval [80.2%; 91.0%]) or meeting with friends (64.5% [65.7–71.6%]). Despite the fact that only about 47.1% [39.2%; 55%] felt well informed about measures against infection, adherence to COVID-19 countermeasures was generally high: 83.9% [77.3; 88.8%] claimed to have worn face masks always/nearly always; social distancing was performed always/nearly always by 58.7% [50.8%; 66.2%]; and hand hygiene was conducted by 64.5% [56.7%; 71.6%] of participants. None out of *n* = 25 tests from unvaccinated subjects was positive for anti-SARS-CoV-2-S-antibodies. Psychiatric comorbidity and educational degree were not statistically significantly associated with attitudes and compliance, except that patients with lower education felt relatively worse informed.

**Conclusion:**

Self-reported adherence to recommended non-therapeutic intervention strategies and vaccination rates were similar to the German general population. Provision of more health-related information tailored to OMT patients appears necessary.

## Introduction

Compared with the age-matched general population, patients in opioid maintenance treatment (OMT) exhibit a higher propensity for an impaired health. Accordingly, the prevalence of comorbid conditions such as cardio-vascular, respiratory, and infectious diseases (e.g., hepatitis B or C) is high (1–3). This increases the risk of severe courses of infectious diseases (4, 5). Given these vulnerabilities, OMT patients (OMTP) would do well to adopt behavioral strategies that reduce the risk of acquiring infections, for example by social distancing or vaccination.

Compared with the general population vaccination rates of illicit opiate drug users or people who inject drugs (PWID) are higher for hepatitis A virus and hepatitis B virus (6, 7), with hepatitis B vaccination programs having shown to be useful in increasing vaccination levels (8), reaching completion rates of up to 70–80% (9–12). Nevertheless, high proportions from these target groups have still not been vaccinated against HBV (6, 7, 13, 14). Influenza vaccination rates of PWID may be lower (5, 15) or similar (7) to those observed in the general population (5).

Regarding COVID-19, studies focusing on OMTP showed considerable variations regarding vaccine hesitancy during the first year of the pandemic: studies from Australia, USA, and Norway showed 20–40% rates of self-reported vaccination rejection (16–18), whereas studies from France, Spain, Australia, USA, and Mexico showed hesitancy in >70% (19, 20), 49% (15), 38% (21), and 38% (22), respectively. What these studies have in common is that vaccination completion rates in PWID were much lower than the rates that were observed in the surrounding general adult population.

Since the early days of the COVID-19 pandemic, health authorities and governmental institutions recommended a number of behaviors to reduce the likelihood of infection and virus dissemination in the population, including thorough hand washing and disinfection, social distancing, use of face masks as well as isolation in case of COVID-19-like symptoms. In Germany, the use of masks was mandatory both in public indoor situations as well as in public transportation.

Empirical findings regarding how PWID or opioid addicts in drug treatment follow such recommendations are sparse. In an early study, conducted from April to June 2020 in Baltimore, USA, high proportions of former or current PWID reported to be well aware of strategies to reduce infection risks, nevertheless, adherence to social distancing was sub-optimal, in particular when they ventured out to acquire illicit drugs (23). During the same period, Norwegian drug treatment clients reported a generally high (80–90%) compliance with COVID-19 recommendations, including SARS-CoV-2 testing upon onset of COVID-19-like symptoms (17). Conversely, daily non-medical opioid users from the north-eastern US region reported lower rates of COVID-19 avoidance behavior compared to non-users (24). In late 2020/early 2021, about 86% of current drug injectors from both San Diego, USA, and neighboring Tijuana, Mexico, reported wearing a face mask, while the other protective behaviors presented (social distancing, self-isolation, and increased hand hygiene) were carried out only by a minority (25). During mid-2020, most patients from OMT clinics in Connecticut, USA reported to comply with social distancing, although only 60% were able to isolate themselves at home most of the time (26).

Overall, PWID seemed to be aware of countermeasures against infections but most empirical studies only dealt with the early phase of the COVID-19 pandemic. There is a lack of empirical studies focusing on OMTPs during later pandemic stages, and their compliance levels regarding behavioral traits reducing the likelihood to acquire SARS-CoV-2 infections.

## Methods

### Setting

The present cross-sectional study was carried out in two outpatient OMT facilities associated with the local academic psychiatry hospital (LVR-Universitätsklinik; Essen, Germany). The city of Essen is part of the Rhine-Ruhr metropolitan area in the Western part of Germany, an area with about 600,000 inhabitants comprising an estimated number of 1,200 opioid dependent subjects, approximately 600 of which enrolled in OMT programs, and about 180 of them being treated in the two contributing OMT facilities.

### Recruitment

The study started in May 2021. All patients currently in OMT in the outpatient facilities were considered for participation. Exclusion criteria were insufficient capacities to express themselves in German or a current diagnosis of a florid psychosis. Eligible patients were informed about the study, and all patients who agreed to be recruited provided written informed consent prior to the interviews and/or tests.

### Assessments

The interviews were conducted in person by one interviewer (proficient medical student T.K.). The standardized interview included basic socio-demographic information (age, sex, migration background, school education, income sources, and living arrangements) and the number of days during the previous 30 days with a reported intake of nicotine, heroin, cocaine, cannabis, alcohol, benzodiazepines, amphetamines, and gabapentinoids. The format of these questions and answers was adapted from the German version of the European Addiction Index (27).

Regarding the current pandemic, using questions with a closed answering format patients were asked whether they had ever undergone any form of SARS-CoV-2 testing, whether they had experienced any symptoms from a list of COVID-19-like symptoms, and whether they had ever been in quarantine (independent from being personally infected). Furthermore, they were asked how many days during the previous 30 days they had engaged in outdoor activities (e.g., using public transport, meeting with friends etc.), and how many personal contacts they had during the previous week. Furthermore, patents indicated their degree of agreement (on a five-point scale from “totally agree” to “totally disagree”) to several statements concerning how well they are aware of available information dealing with COVID-19 and the corresponding countermeasures, and how much they complied with public health measures, typically abbreviated in Germany as “AHA” rules (social distancing, hygiene, and wearing a protective mask; [in German: Abstand, Hygiene, Alltagsmaske or Alltag mit Maske]). Patients were also asked whether they had been vaccinated against COVID-19 or if they intended to do so. Finally, on a four-point rating scale, they indicated their degree of agreement with nine statements about their personal perceptions of non-pharmacologic intervention strategies against COVID-19 (for details see Results section). The recruited subjects’ current psychiatric and medical diagnoses were retrieved from their medical files. In unvaccinated patients, blood samples were tested for anti-SARS-CoV-2-S-antibodies to assess previous infection events.

We report proportions for the answers given and for results of laboratory tests, together with normally approximated 95% confidence intervals, except if proportions were below 10% or higher than 90%, in which case the Wilson score interval with continuity correction was used (28), or in case of zero frequencies, when the rule of three [3/n rule (29)] was used to determine the upper limit of the confidence interval.

Comparisons between independent groups with respect to ordinal scaled variables were carried out using Mann–Whitney U-test, comparisons with respect to categorical variables using Chi^2^ tests, and associations between ordinal scaled variables were analyzed using the Spearman rank coefficient rho.

### Ethics approval

The study was approved by the Ethics Committee of the Medical Faculty of the University of Duisburg-Essen, Germany (21-10093-BO).

## Results

Interview data were collected between May 29th and October 10th, 2021. During this period, 195 patients were treated in the two participating clinics. Among these, 10 were ineligible due to language barrier issues and 30 refused to participate. Hence, 155/185 (84%) eligible patients were included.

### Patient characteristics

Patients here included were on average 47 years old, 73.5% were male, the proportion of participants who were foreign born or whose parents were foreign born was 26.8%, 23.9% had no graduation, 33.5% had left school with a certificate of secondary education, 40% had higher school degrees. Less than one out of four patients indicated to be currently employed, and about half of them were living in a one-person household (see Table 1).

**Table 1:** Patient characteristics.

**Sex**	
Female	40 (25.8%)
Male	115 (74.2%)
**Age**	
Minimum-Maximum	19–71
Mean (SD)	47.1 (9.4)
Age > = 60 years	14 (9.9%)
**Migrant history**	
One parent foreign born	13 (8.4%)
Both parents foreign born	27 (17.4%)
Both parents born in Germany	115 (74.2%)
**Graduation**	
University entrance qualification	22 (14.2%)
Medium secondary school graduate	40 (25.8%)
Lower secondary school graduate	52 (33.5%)
Other	4 (2.6%)
None	37 (23.9%)
**Living conditions**	
Living alone	77 (49.7%)
One roommate	35 (22.6%)
Two or more roommates	26 (16.8%)
Residential home	13 (8.4%)
Homeless	4 (2.6%)
**Employment; previous 6 months**	
Employed full time or part time	37 (23.9%)
Unemployed	92 (59.4%%)
Retired	13 (8.4%)
Not specified	13 (8.4%)

Compared with the study group, patients who were not included had a similar mean age (46 years), were more often male (85.0%), more often had a migrant background (47.5%, as language problems could affect here only migrants and were an exclusion criterion) and showed similar educational attainment (15% no graduation, 47.5% a certificate of secondary education, and 37.5% higher degrees).

Nearly 90% of the study group were tobacco smokers (see Table 2). In addition to their maintenance medication, 63% had consumed one or more psychotropic substances during the previous 30 days, and 56% had at least one additional substance dependence diagnosis concurrent with their opiate dependence clinical condition. About 40% of the recruited subjects presented with a psychiatric diagnosis. For nearly 80%, a medical diagnosis was recorded, the most frequent (49%) being a hepatitis C infection, although for 29% of patients no laboratory test for hepatitis C had been made available. Medical factors known to predispose for more severe courses of COVID-19 such as obesity, chronic obstructive pulmonary disease (COPD), liver disease, diabetes mellitus, lung emphysema or cancer (30) were identified in 39% of patients.

**Table 2:** Somatic and mental health.

**Most frequent somatic comorbidity diagnoses (with ICD code)**	
No diagnosis	33 (21.3%)
Hepatitis C infection (B18.2)	76 (49.0%)
Obesity (E66)	38 (24.5%)
COPD (J44.99)	15 (9.7%)
Essential hypertension (I10.90)	15 (9.7%)
Asthma bronchiale (J45.9)	11 (7.1%)
HIV infection	7 (5.5%)
Diabetes mellitus (E11.80)	5 (3.2%)
Liver cirrhosis (K74.6)	5 (3.2%)
**Smoking**	
Daily cigarette smoker	135 (87.1%)
Daily e-cigarette smoker	6 (3.9%)
Non-daily smoker	2 (1.3%)
Nonsmoker	12 (7.7%)
**Psychotropic substance use (last 30 days)**	
None	57 (36.8%)
Alcohol	48 (31.0%)
Cannabis	58 (37.4%)
Heroin	35 (22.6%)
Cocaine	29 (18.7%)
BZD	23 (14.8%)
Amphetamines	12 (7.7%)
Gabapentinoids	6 (4.9%)
**History of a substance-related diagnosis (except opiate or nicotine dependence)**	
None	68 (43.9%)
One or more	87 (56.1%)
**History of a psychiatric diagnosis**	
None	94 (60.6%)
F2 schizophrenia	8 (5.2%)
F3 affective disorders	41 (26.5%)
F4 anxiety and stress disorders	15 (9.7%)
F6 personality disorders	8 (5.2%)
F9 early onset disorders	3 (1.9%)

### Daily activities

**Figure 1:**
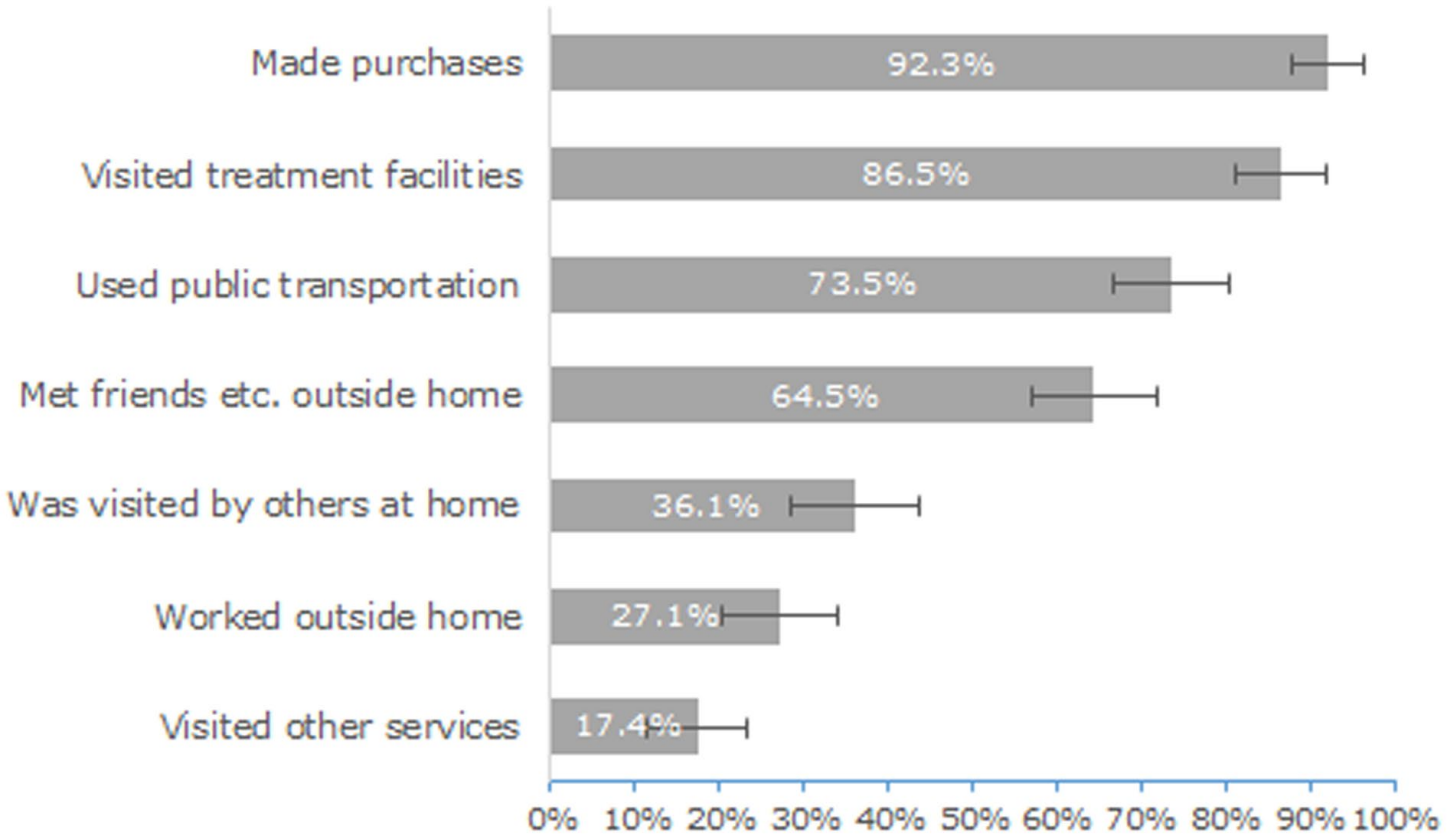
Proportion of patients (with 95% confidence interval) who had engaged in several outdoor activities during the 7 days before the interview.

Only 8.4% [95% C.I. (4.7%; 14.2%)] of the patients had spent the 7 days prior to the interview alone at home, whereas 63.2% [55.6%; 70.8%] had been outside and/or at home with others on a daily basis. As shown in Figure 1, 92.3% [88.1%; 96.5%] reported of having ventured outside home to carry out a range of activities - not necessarily drug-related - during the previous week. In addition, 86.5% [81.1%; 91.9%] attended addiction treatment facilities, for a median of 3 days a week.

### The COVID-19 history of patients

At the time of the interview, half of the group had already received at least one vaccination (see Table 3). Among the non-vaccinated individuals, one third planned a vaccination, while the remaining strongly opposed to be vaccinated. During the course of the study, 25 out of 78 unvaccinated patients donated a blood sample in the context of their routine medical examination, but none of these tests showed a positivity for anti-SARS-CoV-2-S-antibodies [95% C.I. (0%; 12.0%), according to the 3/n rule]. Only 7 out of 155 patients reported symptoms/signs that might have been indicative for a SARS-CoV-2 infection. Most (86.5%) patients have not been in quarantine up to the time of investigation.

**Table 3:** Patients’ history of COVID-19 infection.

Self-reported COVID test results (type of test not specified)	*n* (%)	95% confidence interval
Negative	111 (71.6%)	64.5–78.7
Positive (without symptoms)	1 (0.6%)	0.1–3.6
No tests	43 (27.7%)	21.3–35.3
**Subjective assessment: ever been ill from COVID-19**		
Yes	7 (4.5%)	2.2–9.0
Unsure	9 (5.8%)	3.1–10.7
No	139 (89.7%)	83.9–93.5
**Vaccinated against SARS-COVID**		
Once	51 (32.9%)	26.0–40.6
Twice	26 (16.8%)	11.7–23.4
No	78 (50.3%)	42.5–58.1
**Ever been in quarantine**		
No	134 (86.5%)	80.2–91.0
Yes, during in-patient treatment	1 (0.6%)	0.1–3.6
Yes, because of contact with infected persons	5 (3.2%)	1.4–7.3
Yes, because of residential home policy	13 (8.4%)	5.0–138
Yes, voluntarily	2 (1.3%)	0.4–4.6
**Positive attitude toward being vaccinated**		
Already vaccinated	77 (49.7%)	41.9–57.5
Yes, regardless of the vaccine	27 (17.4%)	12.3–24.2
Yes, but it depends on the vaccine	23 (14.8%)	10.1–21.3
Do not know	9 (5.8%)	3.1–10.7
No	19 (12.3%)	8–18.4

### COVID-19; patients’ general knowledge and adopted levels of personal protection

**Figure 2:**
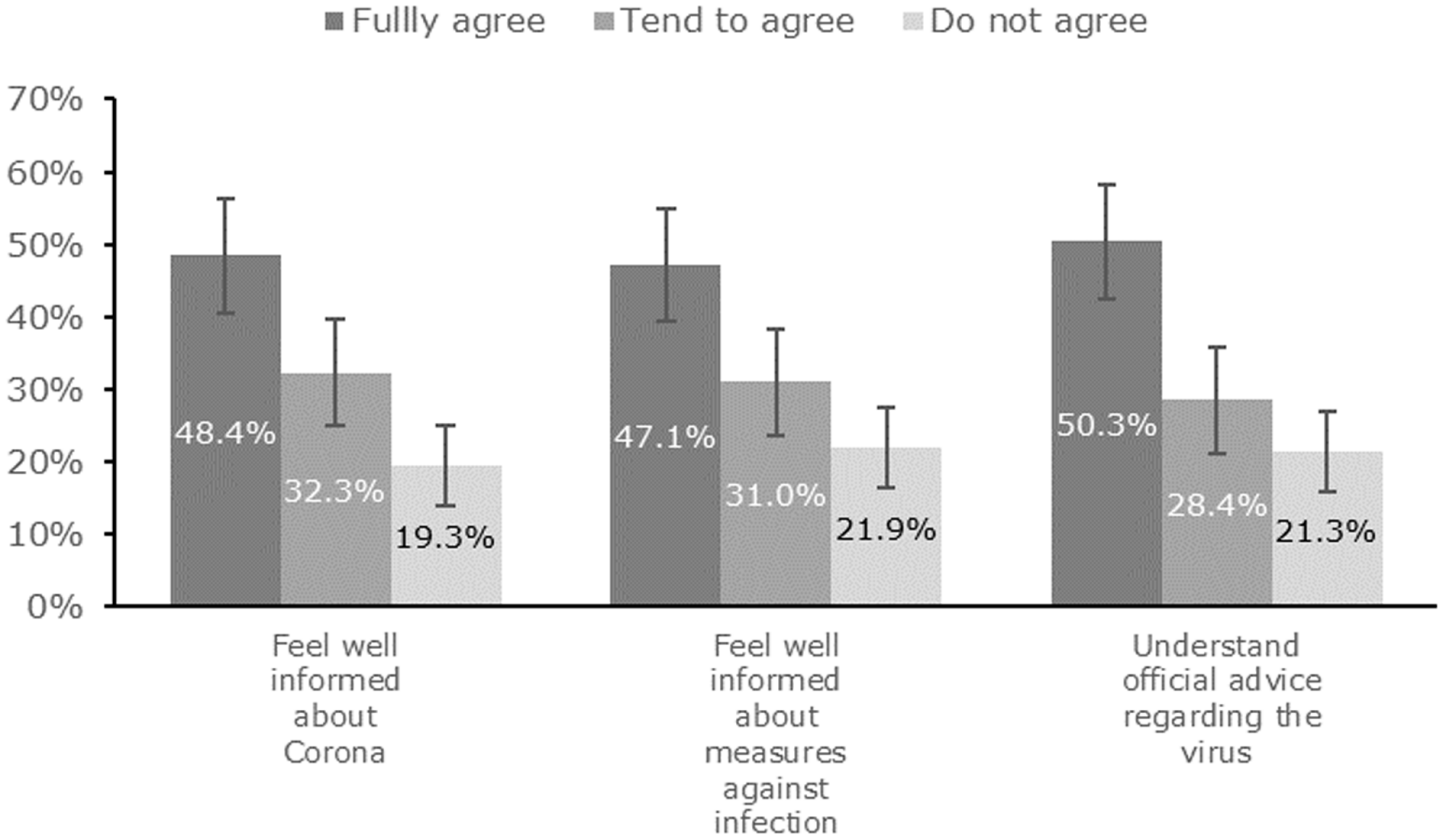
Effects of official safety communication on the individual patient (with 95% confidence intervals).

**Figure 3:**
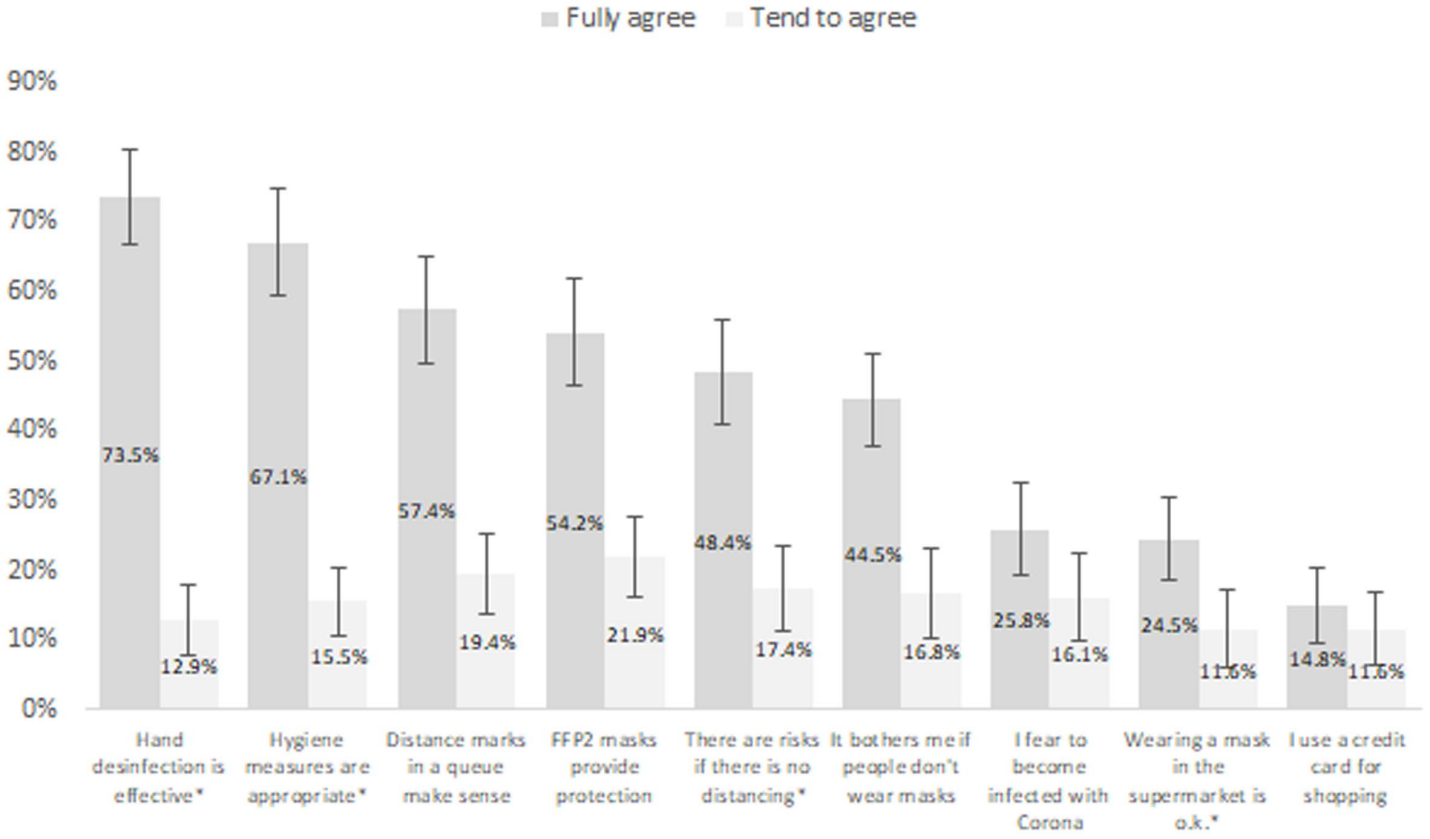
Proportion of patients (with 95% confidence interval) who agreed with statements concerning meaningfulness and effectiveness of protective measures. Missing percentages from 100%: “tend to disagree” or “disagree fully.”

**Figure 4:**
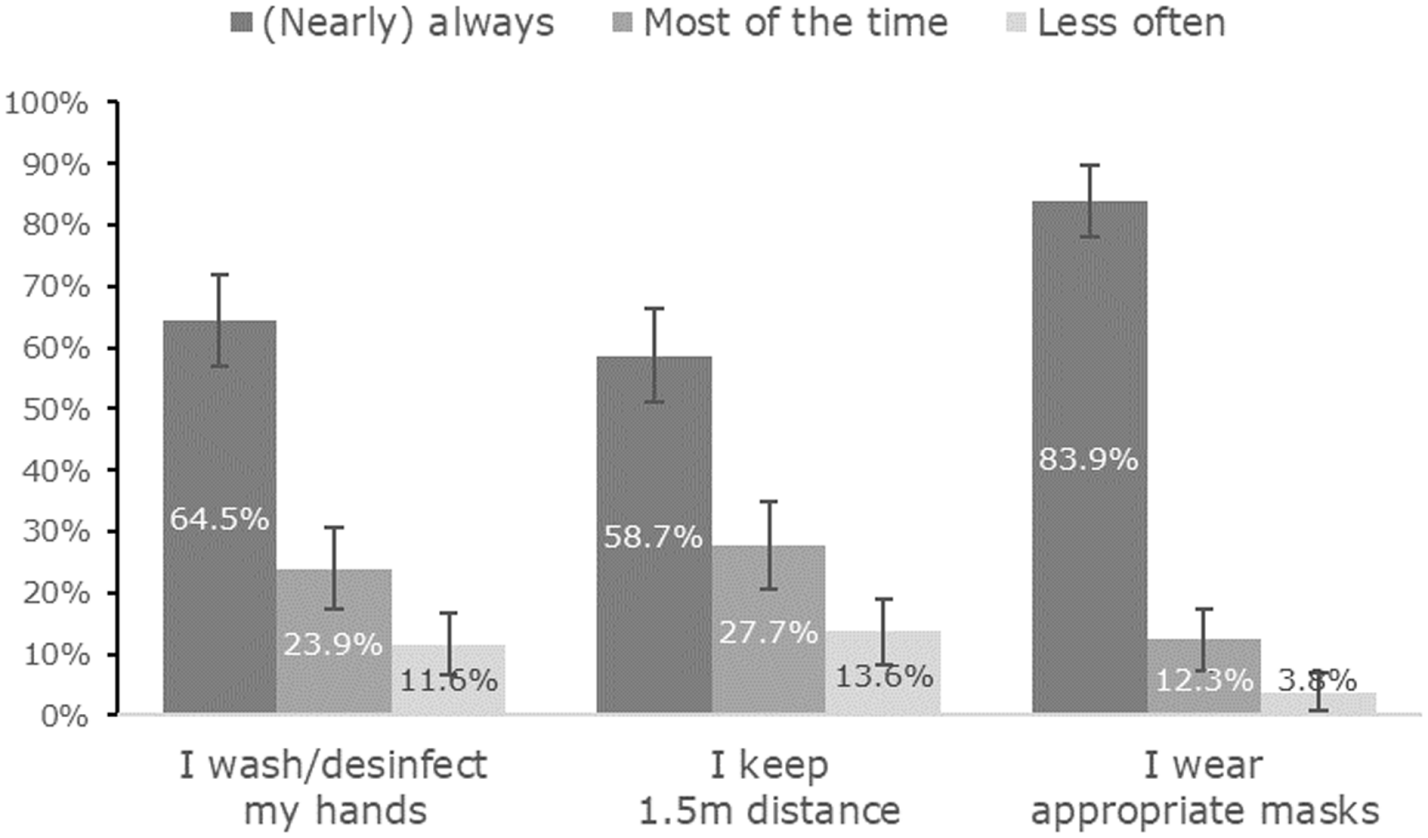
Compliance rates (with 95% confidence intervals) with safety recommendations regarding COVID19.

Only 48.4% [95% C.I. (40.9%; 56.7%)] of patients indicated that they felt adequately informed regarding the virus and the corresponding countermeasures (see Figure 2). Most patients thought that disinfection/hygienic and protective measures were meaningful and effective (see Figure 3): 73.5% [66.6%; 80.5%] fully agreed that hand disinfection was effective, 67.1% [59.7%; 74.5%] that hygiene measures were appropriate, 57.1% [49.6%; 65.2%] that distance narks in a queue make sense, and 54.2% [46.4%; 62.0%] that FFP-2 masks provide protection. A clear majority (58.1%) [50.3%; 65.9%] denied fear of getting an infection. Patients’ self-reports indicated a high degree of compliance with recommended protective behaviors (see Figure 4): 83.9% [78.1%; 89.8%] wore a protective mask (nearly) always, while adoption of hand hygiene ([nearly] always 64.5% [57.0%; 72.0%]) and social distancing measures ([nearly] always 58.7% [50.9%; 66.5%]) were less frequent (see Figure 4).

### Associations with psychiatric comorbidity and with educational attainment

We grouped the patients into those with diagnoses of psychiatric comorbidity (PCD, *n* = 60), and those without (NPCD, *n* = 94). Using a sum score of the 9 attitude measures, the median was 19 points for the comorbid group, and 18 for the non-comorbid group (*p* = 0.53 [Mann–Whitney U-test]). The statement that they felt well informed about Corona was fully agreed with by 43.3% (PCD) vs. 45.3 (NPCD) (*p* = 0.42, U-test for comparison on the 4-point scale), that they felt well informed about measures against Corona by 48.3% vs. 46.3% (*p* = 0.93), and that they understood official advice by 51.7 vs. 49.5% (*p* = 0.92). Wearing a mask (nearly) always was reported by 88.1% vs. 81.1% (*p* = 0.23, U-test for comparison on the 4-point scale), keeping social distance by 57.7% vs. 60.0% (*p* = 0.61), and hand disinfection by 58.3% vs. 69.4% (*p* = 0.18). In addition, both groups had similar vaccination rates (PCD 48.3%, NPCD 49.5%, *p* = 0.87 [Chi^2^ test]).

The Spearman rank correlation of educational attainment with the attitudes sum score was rho = −0.03 (*p* = 0.73), with “feeling well informed about Corona” (4-point scale) rho = 0.14 (*p* = 0.093), with “feeling well informed about measures against Corona” rho = 0.16 (*p* = 0.045), and with “I understand official advice,” rho = 0.23 (*p* = 0.003). Regarding the compliance measures, with hand disinfection the correlation was rho = −0.13 (*p* = 0.12), with social distancing, rho = −0.04 (*p* = 0.59), and with wearing a mask rho = 0.03 (*p* = 0.73).

To conclude, psychiatric comorbidity showed very little and statistically not significant associations with attitudes, feeling informed, or self-reported compliance, while level of education also was not associated with attitudes or compliance, but showed small to medium, and statistically significant, associations with feeling informed.

## Discussion

The present study describes both attitudes and self-reported compliance with recommended protective measure against SARS-CoV-2 infections and COVID-19 disease, including vaccination, in a sample of OMT patients. Typical subjects in our cohort were middle-aged males, living alone, with medium to high educational attainment levels in about 40% of cases, and high unemployment rates. For various reasons, almost all patients ventured outside their home during the week preceding the interview, including their routine attendance at the maintenance treatment facility. Apart from an addiction disorder, approximately 80% of recruited subjects presented with an additional medical disorder. In half of these cases, this comorbidity is known to increase the risk of severe COVID-19 disease courses.

Half of the patients felt that they were not adequately informed about the virus or infection avoidance measures, and such feeling was moderately, and statistically significantly, correlated with declining levels of education. Fear of contracting the SARS-CoV-2 infection was reported by only 1/4 of recruited subjects. Accordingly, about 28% had never performed a SARS-CoV-2 test, some 33% showed levels of reluctance in considering an anti-COVID-19 vaccination, including 12% who explicitly opposed the vaccination. Conversely, most patients reported a high degree of compliance with recommended protective behaviors, in particular some 84% reportedly wore face masks always/nearly always.

Present findings shall be interpreted in comparison with surveys dealing with the general German population. Between November and December 2020, a large (*n* = 5,843), well-stratified, general German sample, quantified the levels of compliance toward COVID-19 protective measures on a 7-point scale (e.g., from 1: “does not apply at all” to 7: “applies completely”). In terms of social distancing (1.5 m); hand hygiene, and wearing face masks, some 73% indicated 6 or 7 points (31). A prospective cohort study conducted between January 2020 and June 2021, a German population-based sample (*n* = 10,250) showed levels of compliance toward social distancing in only 48.3% of cases, and hand hygiene in less than 25% of cases. Conversely, the use of face masks was reported to be very frequent (91.5%) (32). Compared to males, women were more likely to engage in protective behaviors and showed higher anti-COVID-19 vaccination rates. Hence, one would conclude that self-reported adherence levels of OMT patients to COVID-19-related advice and recommendations were similar to those from the German general population.

Comparisons of the vaccination rate with the general population are difficult to make, since this rate increased dramatically during the data collection period between May and October 2021. Patients interviewed during May (*n* = 31), showed a 22.6% vaccination rate, including *n* = 1 with basic immunization (two vaccinations), while those interviewed from mid-September to mid-October (*n* = 19) reported a 73.7% vaccination rate (including 31.6% with already basic immunization). The basic immunization rate appears markedly lower than in the general population aged 18–59 years, which was 17% by the end of May 2021, 54% by the end of July, and 70% by the end of September (33), but it may be assumed that there was only some delay of the vaccination, compared with the general population, and not a rejection of it. A lower vaccination rate in the patient group had to be expected anyway, given the over-representation of males (74% here vs. 49% in the general population), a factor predictive of more vaccination hesitancy (34–36).

The very few published studies dealing with OMT patients are consistent with our findings and suggest overall high levels of self-reported compliance with anti-COVID-19 behavioral recommendations (17, 26). Patients appear to regard themselves as being part of a high-risk group, which may result in a motivation for adherence to advice and recommendation that minimize the risk of infection. It is a reason of concern, however, that a significant proportion of patients did not feel adequately informed about the virus and countermeasures that mitigate virus transmission and dissemination. This in itself is a factor being associated with decreased levels of compliance to official recommendations (34, 37). One protective factor could have been here the regular and often daily contact with the OMT clinic, where the SARS-CoV-2/COVID-19 countermeasures were enforced during the pandemic, which may have raised the awareness. In addition, OMT facilities offered an easy access to vaccination programs.

Of further interest are the low self-reported, and laboratory-confirmed, SARS-CoV-2 infection rates identified in the subgroup of unvaccinated patients. Although drug abusing patients often show unsafe/unhealthy behavior (e.g., sharing of non-sterile equipment, consumption of contaminated substances, or overdosing), this does not necessarily mean that they may refrain from adopting health-preserving behaviors, either related or unrelated to their substance use. Harm-reduction measures such as needle sharing programs indeed postulate that their target group is willing and proficient to adopt healthy behaviors (38). Finally, the huge differences in vaccination rates reported for different countries such as the France, USA, Spain, and Mexico [e.g., (19–22)], may suggest a relevant influence of context factors such as the characteristics of the national/local health system.

## Limitations and conclusions

The present study was not carried out to provide evidence for the effect of preventative measures on infection rates, and hence extensive laboratory measurements were not here made available. One could wonder about the validity of self-reported data, with patients having possibly answered in a way that they assume to be socially desirable, e.g., indicating higher degrees of compliance to anti-COVID-19 recommendation and advice. While this concern cannot be ruled out completely, the pattern of results also included socially “undesirable” answers from many of the interviewees as well. Importantly, data from the general population could have presented with the same bias.

Taken together, we suggest providing better and more suitable health-related information to vulnerable drug dependent OMT subjects.

## Data availability statement

The raw data supporting the conclusions of this article will be made available by the authors, without undue reservation.

## Ethics statement

The studies involving humans were approved by Ethics Committee of the Medical Faculty of the University of Duisburg-Essen, Germany. The studies were conducted in accordance with the local legislation and institutional requirements. The participants provided their written informed consent to participate in this study.

## Author contributions

NS: Conceptualization, Supervision, Writing – review & editing. MS: Data curation, Writing – original draft, Writing – review & editing. TK: Data curation, Writing – review & editing. MT: Formal analysis, Writing – review & editing. UB: Writing – review & editing. FS: Writing – review & editing.

## Conflict of interest

The authors declare that the research was conducted in the absence of any commercial or financial relationships that could be construed as a potential conflict of interest.

## Publisher’s note

All claims expressed in this article are solely those of the authors and do not necessarily represent those of their affiliated organizations, or those of the publisher, the editors and the reviewers. Any product that may be evaluated in this article, or claim that may be made by its manufacturer, is not guaranteed or endorsed by the publisher.
